# Spelling of Medical Words

**Published:** 1893-08

**Authors:** 


BUFFALO MEDICAL AND SURGICAL JOURNAL

A MONTHLY REVIEW OF MEDICINE AND SURGERY.

*EDITORS:*

THOMAS LOTHROP, M. D. - - WM. WARREN POTTER, M. D.

All communications, whether of a literary or business character, should be addressed to the managing editor: 284 FRANKLIN STREET, BUFFALO, N. Y.

VOL. XXXIII. AUGUST, 1893. No. 1.

### SPELLING OF MEDICAL WORDS.

FOR some time past there has been going on in certain circles an agitation looking to reform in the orthography of medical words and terms. The chemists have already successfully met the question by the adoption of simpler endings, and substituting "f" for "ph" in such words as admit of that change. Many medical philologists, scholars, and editors have, for some time past, dropped the use of diphthongs whenever practicable, and otherwise simplified medical spelling in accordance with their own fancy or good sense. Dr. George M. Gould, of Philadelphia, the accomplished editor of the *Medical News*, and the author of two excellent dictionaries, is an earnest laborer in this field, and has signaled his efforts on several occasions, both in writing and by speech. One of his latest efforts was a paper read at Milwaukee, in June, at the meeting of the American Medical Association, in which he deals in detail with the subject. The paper was published in the *Medical News*, and we have taken the liberty to reproduce it in this number of the JOURNAL, and we ask for it a patient examination by all our readers. It is to be hoped that the traditions that have clung to the past, and are not easily shaken off the present, will begin to loosen their hold on the minds of medical writers and teachers to the much-desired simplification of the writing and printing of many cumbersome medical words and phrases.

